# Neurocardiology Update: The Brain–Heart Connection in Multiple Sclerosis—A Narrative Review

**DOI:** 10.1002/hsr2.70607

**Published:** 2025-03-25

**Authors:** Nicoleta Gherghel‐Pavăl, Denis Pavăl, Adina D. Stan, Olga H. Orășan, Adela V. Sitar‐Tăut, Angela Cozma

**Affiliations:** ^1^ 4th Department of Internal Medicine “Iuliu Hațieganu” University of Medicine and Pharmacy Cluj‐Napoca Romania; ^2^ Department of Psychiatry “Iuliu Hațieganu” University of Medicine and Pharmacy Cluj‐Napoca Romania; ^3^ Department of Neurology “Iuliu Hațieganu” University of Medicine and Pharmacy Cluj‐Napoca Romania

**Keywords:** cardiovascular dysfunction, multiple sclerosis, neurocardiology

## Abstract

**Background and Aims:**

While multiple sclerosis (MS) is primarily considered a neurological disorder, mounting evidence suggests a strong association with cardiovascular diseases (CVDs), impacting both disease progression and patient outcomes. This paper aims to raise awareness of this disease association while promoting a clinical‐oriented, multidisciplinary approach that can provide long‐term benefits for these patients.

**Methods:**

A comprehensive literature review was conducted to gather up‐to‐date evidence concerning the incidence and type of CVDs associated with MS, the mechanisms underlying this disease association, as well as the impact on MS progression. Based on this evidence, a neurocardiological approach to MS in clinical practice was proposed.

**Results:**

Past and present research agree on the high rates of arterial hypertension, metabolic syndrome, stroke, and myocardial infarction in people with MS. However, other prevalent comorbidities, such as venous thromboembolism and autonomic dysfunction may be easily overlooked in clinical practice. A complex interplay between genetic predisposition, traditional risk factors, autonomic dysfunction, inflammation, and treatment‐related factors likely plays a role in promoting CVDs in MS. The impact of cardiovascular dysfunction on MS progression ranges from subclinical impairments, such as impaired axonal repairing, to overt physical disability and cognitive dysfunction. This paper proposes a neurocardiological approach to the daily clinical practice of MS patients, comprising general lifestyle measures, comorbidity screening at MS diagnosis, extensive work‐ups for progressive/active forms, and selected autonomic dysfunction screening.

**Conclusion:**

MS is a lifelong disorder that can be associated with a multitude of comorbidities, particularly cardiovascular ones. Along with increased mortality, cardiovascular pathology can adversely affect clinical and radiological‐derived MS outcomes. Thus, surveillance and preventative measures are required for this population.

**Abbreviations**
CABF: cerebral arterial blood flow
CNS: central nervous system
CVDs: cardiovascular diseases
CVR: cerebrovascular reactivity
DMT: disease‐modifying treatment
MI: myocardial infarction
MS: multiple sclerosis
T2D: type 2 diabetes

## 1 Introduction

Multiple sclerosis (MS) is the leading cause of non‐traumatic neurological disability in young and middle‐aged adults. Despite being a significant public health issue, its pathogenesis and outcome determinants are poorly understood [1].

Genetic, environmental, and comorbidity‐related factors likely determine the course of MS. Among these, comorbidity particularly appeals to neurologists since it can be preventable or treatable [2]. Recently, the association between MS and cardiovascular diseases (CVDs) has gained significant interest. Retrospective and cohort studies have shown that MS is associated with a higher risk of acute coronary syndrome, myocardial infarction (MI), and stroke [3, 4, 5]. In turn, cardiovascular comorbidity is linked to higher mortality and worse neurological outcomes, such as disability and poor cognitive performance [6, 7].

This intricate interplay opens the way for a neurocardiological approach to MS in clinical practice. In this paper, we highlight this interplay's relevance to neurologists and cardiologists and emphasize novel approaches readily available for inclusion in routine clinical practice.

## 2 The Burden of Cardiovascular Disease in Multiple Sclerosis

Research has found that people with MS have a higher risk of developing CVDs compared to the general population. Some of the comorbidities that have been extensively studied include hypertension, type 2 diabetes (T2D), obesity, hyperlipidemia, metabolic syndrome (MetS), MI, and stroke [8].

While hypertension is the third most common comorbidity amongst MS patients [9], with a prevalence as high as 18.2% [10], the association between MS and T2D is more controversial. Kang and colleagues [11] found that T2D was 1.5 times more prevalent in MS than in controls (8.6% vs. 6.1%). However, a different study reported a similar age‐adjusted prevalence of T2D in MS patients and controls (8.31% vs. 7.62%) [12]. On the other hand, a recent meta‐analysis reported a 5% prevalence of T2D in MS patients as compared to 10% in the general population [13]. These conflicting results underscore the need for further investigation as it could improve long‐term outcomes in MS patients.

The prevalence of obesity among individuals with MS has also shown inconsistency across different studies. While some authors reported an increased prevalence [14], others reported similar [15] or decreased [16] rates. More evidence concerning obesity in MS comes from a recent meta‐analysis showing that the mean waist circumference and body mass index among MS patients were estimated to be 87.27 cm and 25.73, respectively [17].

Most studies have reported an unfavorable lipid profile in MS patients. However, the results differ depending on the disease‐modifying treatment (DMT) type and the cut‐off values for dyslipidemia [8]. According to recent findings from a meta‐analysis, the pooled‐prevalence of dyslipidemia in MS patients is 11.5% [10].

Recent research has addressed the relationship between MS and MetS (comprising abdominal obesity, dyslipidemia, insulin resistance, and vascular dysfunction) [18, 19]. In a cohort of 84 MS patients, 27% were found to have MetS. Moreover, MetS was more prevalent in secondary progressive MS and was linked to disability progression [18].

Furthermore, patients with MS have a higher risk of cardiac, cerebrovascular, and peripheral vascular disease than the general population. Indeed, data from systematic reviews indicate that most well‐designed studies report a high incidence of ischemic stroke, ischemic heart disease, and peripheral artery disease among MS patients compared to the general population [9]. The crude prevalence of ischemic heart disease varied from 0.78% to 22.2% across different studies, with population‐based research indicating a prevalence of 2.5% [9]. The incidence of MI across MS cohorts ranged from 2.36% to 2.75% [9]. In addition, the same systematic review showed a prevalence of 2.4% for peripheral artery disease. A recent multi‐database study [20] confirmed the high risk, reporting a twofold higher rate of peripheral vascular disease in MS patients compared to non‐MS patients and a 2.5‐times greater risk of MI in females with MS compared to those without MS.

In addition, an incidence ratio of 1.7 to 1.96 for stroke was reported across studies [21, 22]. Moreover, the increased risk of stroke remained present for 30 years after the onset of MS [22]. In the multi‐database study, Persson et al. [20] found that the stroke rate among MS patients was elevated compared to non‐MS patients (incidence rate ratio, IRR 2.03) and that the risk was more elevated in women as compared to men (IRR 2.19 and 1.71, respectively). More evidence comes from a recent systematic review and meta‐analysis showing that MS patients have a higher risk of all‐cause stroke, acute ischemic stroke, and intracerebral haemorrhage compared to the general population (RR: 2.55, 2.79, and 2.31, respectively) [10]. Similarly, Palladino et al. [3] found that MS patients had a higher risk of acute coronary syndrome, cerebrovascular disease, and any macrovascular disease. Even after accounting for traditional risk factors like hypertension and dyslipidemia, MS patients still face increased cardiovascular risk, suggesting the presence of distinct underlying mechanisms [4].

MS is also associated with an increased risk of heart failure, particularly in women and younger patients [23]. Research using 3D and speckle tracking echocardiography has shown that both left and right ventricular function are impaired in MS [24].

Moreover, patients with MS are at higher risk of developing venous thromboembolism due to disease‐associated immobility [25]. Studies have shown that the risk of venous thromboembolism is higher in both relapsing‐remitting and progressive forms of MS as compared to the general population [26].

Another well‐known cardiovascular comorbidity with influence on both disease pathogenesis and course is autonomic dysfunction [27]. Various methods have been used to study cardiovascular autonomic modulation in MS, including impedance cardiography, blood pressure and heart rate variability, expiration/inspiration ratio, and tilt test. Data suggests that patients with progressive MS forms have a shift of basal sympathetic‐parasympathetic cardiac modulation towards sympathetic predominance as compared to those with relapsing‐remitting forms or healthy controls. In addition, the same group has decreased parasympathetic cardiac autonomic tone both at rest and in response to deep breathing testing [28]. Additionally, there seems to be a connection between autonomic dysfunction and the stage of the disease. While parasympathetic involvement predominates in advanced disease stages, sympathetic dysfunction is associated with early inflammatory activity [29]. Clinically, high rates of orthostatic hypotension and postural orthostatic tachycardia syndrome have been reported [30, 31]. However, more severe comorbidities related to autonomic dysfunction may be overlooked. Although most of the data comes from case reports, some examples include neurogenic pulmonary edema, takotsubo‐like cardiomyopathy, and sudden death [32, 33, 34, 35]. Thus, increased awareness of autonomic dysfunction presence is essential, especially in progressive or highly active forms of MS.

To summarize, research strongly suggests that individuals with MS have a higher risk of developing CVDs in comparison to the general population. The following section will explore the potential connection between cardiovascular dysfunction and MS.

## 3 Mechanisms Underlying Cardiovascular Dysfunction in Multiple Sclerosis

There is limited data on how cardiovascular dysfunction occurs in MS. However, a complex interplay between genetic predisposition, traditional risk factors, autonomic dysfunction, inflammation, and treatment‐related factors likely plays a role.

According to a recent Mendelian randomization study, there is evidence suggesting that MS is causally associated with CVDs [36]. Results showed that genetic predisposition to MS increases the risk of coronary artery disease, MI, heart failure, and ischemic stroke. Using similar methods, another study provided evidence of a genetic correlation between obesity and MS by identifying 39 shared‐risk single nucleotide polymorphisms [37].

It is reasonable to assume that the high prevalence of traditional risk factors such as smoking or reduced physical activity is responsible for a proportion of the cardiovascular burden [22, 38]. However, the increased risk that persists even after accounting for these factors suggests distinct underlying mechanisms [4], which we will discuss further.

Studying vascular changes in the central nervous system (CNS) can provide valuable insights into the mechanisms underlying CVD in MS. Recent research has focused on better understanding cerebral hemodynamics by assessing dynamic cerebral autoregulation and cerebral vasoreactivity (CVR) using transcranial Doppler ultrasound [39]. Data suggests that the global cerebral hypoperfusion and impaired CVR reported in MS patients may be related to a widespread endothelial dysfunction leading to impaired autoregulation [39, 40, 41].

Several factors, such as inflammation, oxidative stress, and hyperhomocysteinemia, may contribute to endothelial dysfunction in MS [42, 43]. Indeed, an increased level of systemic and CNS oxidative stress has been reported in MS patients as compared to healthy individuals [44]. These alterations and vascular inflammation can lead to endothelial dysfunction and arterial remodeling, thus promoting atherosclerosis [24]. Moreover, CNS inflammation can lead to the release of catecholamines, which may affect cardiovascular function [45].

Research has emphasized the importance of the autonomic nervous system in immune system regulation, which could influence disease progression in conditions such as MS [27]. In MS, demyelinating lesions affecting specific structures can be a potential mechanism of cardiovascular dysfunction. Cardiovascular autonomic dysregulation is presumed to arise from midbrain lesions, although other structures, such as the insula or limbic system, may also contribute [46, 47]. Indeed, signs of cardiovascular autonomic dysfunction, such as reduced heart‐rate variability and decreased blood pressure reactions, were found to be correlated with the MRI lesion load [47]. The association between disease activity duration and sympathetic dysfunction suggests that immune dysregulation could negatively impact the autonomic system [29].

Lastly, medication used for MS may contribute to cardiovascular dysfunction. Thus, the use of glucocorticoids for acute relapses is associated with an increased risk of CVDs, such as MI, heart failure, and cerebrovascular disease [48]. Also, while not commonly used today, mitoxantrone is well‐known for its cardiotoxicity [49]. Although rarely, novel agents have been shown to induce cardiac events, for example, bradycardia and atrioventricular blocks with fingolimod, hypertension with teriflunomide, coronary arterial disease, atrial fibrillation and heart failure with ocrelizumab [50, 51, 52].

To summarize, the cardiovascular burden in MS may arise from vascular changes, strategic demyelinating lesions, immune system dysregulation, and treatment‐related effects. In turn, cardiovascular dysfunction can worsen the prognosis of MS, contributing to disease progression. In the upcoming section, we will delve into the specific aspects of this cascading effect.

## 4 The Impact of Cardiovascular Dysfunction on Multiple Sclerosis Progression

Several studies have examined the impact of cardiovascular comorbidities on MS progression. The range of effects spans from subclinical impairments, such as impaired axonal repairing, to overt physical disability and cognitive dysfunction. In addition, CVD significantly worsens MRI‐derived disease outcomes [7].

At a fundamental level, CVDs may contribute to the neuroinflammatory and degenerative processes in MS by reducing cerebral arterial blood flow (CABF) [53]. Hypoperfusion may promote mitochondrial failure, oxidative damage, and impaired axonal repair [54]. Using 7 T MRI volumetric and ^31p^ MR spectroscopic imaging techniques, a recent study has found that MS patients with vascular disease risk factors have a lower brain ATP than those without. This finding may indicate an early sign of mitochondrial dysfunction induced by altered vascular health [55]. Another study supporting this theory showed that a more effective cerebral autoregulation is associated with better structural brain parameters, such as larger gray matter and lower white matter hyperintensity volumes [39]. Moreover, reduced CABF has been directly correlated with adverse clinical outcomes such as cognitive dysfunction or physical disability [39, 56, 57]. Besides, conditions known to increase the inflammatory burden, such as T2D and obesity, may further disrupt the blood–brain barrier, enabling immune cells to enter the brain [58].

At a clinical level, the overall presence of cardiovascular comorbidities heightens relapse rates and negatively impacts disability status. Petruzzo et al. [59] studied the impact of CVDs on both MS relapses and disability progression by utilizing the Framingham risk score. The results showed that a one‐point score increase was associated with a 31% higher risk of relapses and a 19% higher risk of reaching a score of 6.0 on the Expanded Disability Status Scale (i.e., ambulatory impairment requiring a cane to walk 100 m). Another study found that having vascular comorbidity at any point during the disease increased the risk of disability progression. Furthermore, the risk depended on the number of vascular comorbidities at MS diagnosis. A single comorbidity increased the risk of early gait disability by 51%, while two increased it by 228% [60]. Recently published data provides further information on the connection between comorbidities and disease activity in MS patients, defined as confirmed relapse, disability worsening, or new lesions on MRI. In a meta‐analysis of 17 clinical trials comprising almost 17,000 MS patients, the presence of two or more cardiometabolic comorbidities was associated with an increased hazard of disease activity (adjusted hazard ratio: 1.21) [61].

In addition, several distinct cardiovascular comorbidities have been demonstrated to worsen MS prognosis. Among autonomic symptoms, orthostatic intolerance has been linked to reduced quality of life and fatigue [62]. Some researchers have suggested that cardiac autonomic dysfunction may contribute to the high incidence of falls in MS patients due to fatigue and reduced blood flow to the brain [63].

Several studies have found that plasma homocysteine levels are elevated in MS regardless of the stage or activity of the disease [64, 65]. Among these patients, high levels of homocysteine have been independently associated with cognitive impairment [65].

On the other hand, the presence of cardiovascular comorbidities has been linked to poor response to DMT or the need to escalate treatment. In their study, Petruzzo and colleagues found that each one‐point increase in the Framingham risk score was linked to a 62% higher chance of DMT escalation [59]. Indeed, research has shown that overweight patients are less likely to experience no evidence of disease activity when treated with interferon‐beta compared to those with a normal body mass index [66].

Finally, cardiovascular comorbidities are also reflected in different radiological outcomes. Patients with MS who have one or more cardiovascular risk factors have increased lesion burden and higher degrees of brain atrophy [67].

**Figure 1:**
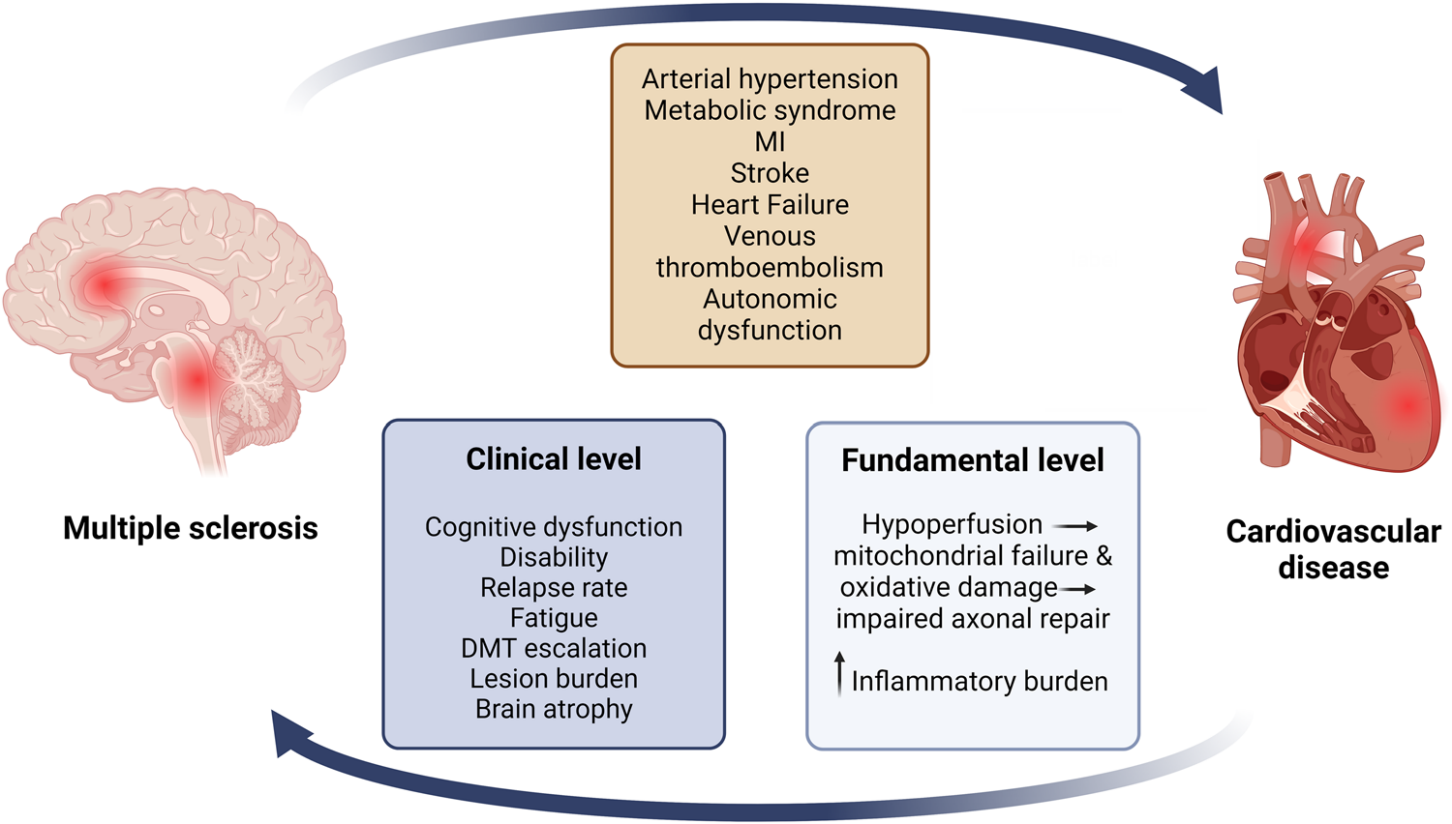
The brain–heart connection in multiple sclerosis. CVDs, cardiovascular diseases; DMT, disease‐modifying treatment; MI, myocardial infarction; MS, multiple sclerosis. MS and CVDs share a bidirectional relationship. MS increases the risk of various CVDs, including hypertension, metabolic syndrome, MI, and stroke. Conversely, CVD negatively affects MS progression at both fundamental and clinical levels. At a fundamental level, CVD may lead to cerebral hypoperfusion, promoting mitochondrial failure, oxidative damage, and impaired axonal repair. Conditions such as type‐2 diabetes and obesity may further disrupt the blood–brain barrier, enabling immune cells to enter the brain, thus increasing the inflammatory burden. On a clinical level, the presence of cardiovascular comorbidities leads to worse cognitive function and fatigue, worse MRI outcomes, increased relapse rates, and the need for more aggressive treatment.

Thus, there is consistent evidence that the presence of CVDs negatively impacts MS progression. Therefore, preventing and treating these comorbidities could result in better neurological outcomes. The complex interplay between MS and CV disease is outlined in Figure 1.

The following section will explore different methods for implementing neurocardiology in daily clinical practice.

## 5 A Neurocardiological Approach to Multiple Sclerosis in Clinical Practice

According to the 2021 European Society of Cardiology Guidelines, it is advisable to evaluate the cardiovascular risk in individuals with inflammatory conditions and provide similar interventions to high‐risk general populations [68]. Thus, patients with MS may benefit from a multidisciplinary approach.

A recent large population‐based study found that despite having more vascular risk factors, patients with MS are less likely to receive treatment. The study revealed that among patients with hypertension and T2D, the usage of specific drugs was 66% and 56% lower, respectively, in MS patients compared to controls. Additionally, the use of lipid‐lowering treatment was 63% lower in the MS group [69].

**Figure 2:**
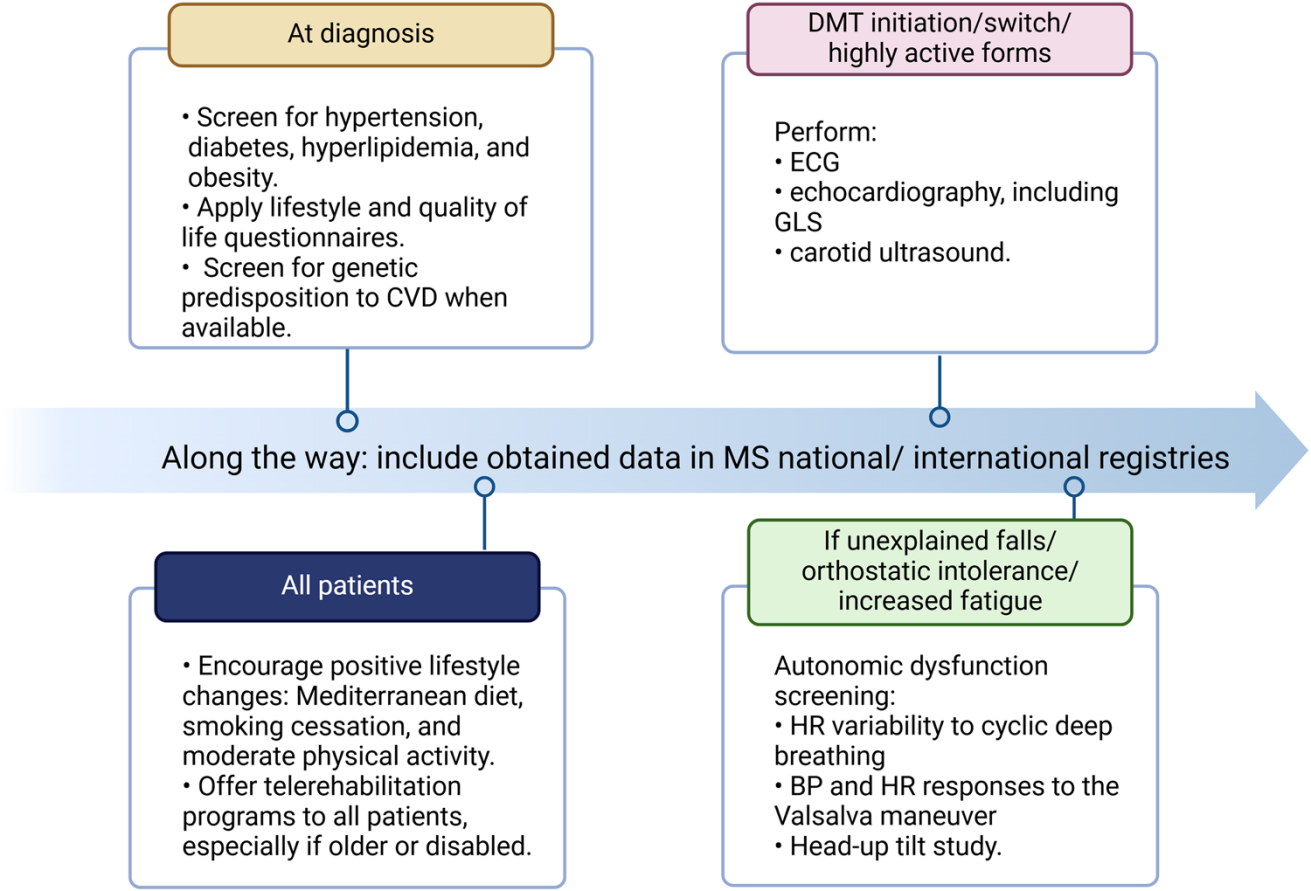
A neurocardiological approach to multiple sclerosis in clinical practice. BP, blood pressure; DMT, disease‐modifying treatment; ECG, electrocardiography; GLS, global longitudinal strain; HR, heart rate; MS, multiple sclerosis. We propose a framework for approaching cardiovascular comorbidity in MS patients. All patients should be screened at diagnosis for both lifestyle habits and cardiovascular comorbidities, such as hypertension and diabetes. In addition, positive lifestyle changes should be encouraged. A more extensive cardiovascular workup may be used in specific situations, such as at DMT initiation or in aggressive disease forms. Specific tests for detecting autonomic dysfunction may be applied if patients present unexplained falls, increased fatigue, or signs of orthostatic intolerance.

Being proactive in monitoring and preventing cardiovascular comorbidities can have long‐term benefits for patients with MS. First, this approach could diminish the adverse effects on clinical outcomes, such as cognitive dysfunction and disability. Given the young age of this population, this may prove to be a cost‐effective long‐term strategy. Second, with constant advancements in DMT, prior cardiovascular screening may be mandatory due to increased risk of developing comorbidities such as hypertension and arrhythmia. However, neurologists responsible for MS patients may face challenges in screening for cardiovascular pathology without an established algorithm. Thus, the best approach for detecting and preventing CVDs in MS patients remains uncertain. In Figure 2, we propose a general framework that can be adapted in clinical practice based on clinical judgment and available resources.

The first step would be to estimate patients' quality of life and lifestyle by applying appropriate scales and questionnaires, such as the *Multiple Sclerosis Quality of Life‐54* (MSQOL‐54), the *Lifestyle Questionnaire*, or *Adherence to a Healthy Lifestyle Questionnaire*. Then, patients should be advised to adopt positive lifestyle habits. Neurologists should discourage smoking and physical inactivity, explaining their harmful effects on MS disability progression and the risk for concurrent CVDs. Moreover, a Mediterranean or similar diet should be recommended to lower cardiovascular risk. Since physical exercise could represent a real challenge for older or more disabled MS patients, remotely offered exercise training programs could be offered in these cases. Recently, protocols for such MS patients have been developed and tested, with vascular dysfunction parameters being one of the outcomes [70]. In the upcoming years, home‐based exercises delivered through tailored telerehabilitation programs could represent a better, generalized option for MS patients with fewer costs and positive cardiovascular outcomes.

When establishing an MS diagnosis, patients should be concomitantly screened for hypertension, hyperlipidemia, obesity, and diabetes [71]. Noninvasive electrocardiography, echocardiography, and carotid ultrasound evaluation may help monitor CVD when initiating/switching DMT, or in very active MS forms. Centers equipped with suitable resources could screen for genetic predisposition to CVD to identify which patients would benefit from a more thorough approach.

Given the high prevalence of autonomic dysfunction that can often be overlooked for years, screening with tilt and/or sudomotor tests may be helpful. This screening approach would benefit patients with orthostatic intolerance or frequent falls (not explained by concomitant neurological deficits) or those complaining of increased fatigue. A multidisciplinary team, including a cardiologist, should coordinate the whole process.

Nevertheless, we acknowledge that further research is required to establish the cost‐effectiveness of such protocols in clinical practice. There is also an emergent need to determine which MS patients would be suitable for more extensive workups such as echocardiography and carotid Doppler ultrasound.

Finally, there is no doubt that there is a lot of potential for further research in this area. One possible research direction is to explore whether improving cerebral perfusion can positively impact brain atrophy and cognitive function. The potency, safety, and efficacy of GLP‐1 receptor agonists are also worth studying in this population due to their ability to reduce both vascular risk and neurodegeneration [72]. In addition, assessing the effectiveness of DMT in lowering cardiovascular risk and evaluating the impact of treating cardiovascular comorbidities on MS outcomes can contribute to a better understanding of this disease association. Thus, forthcoming MS registries may provide additional insights into cardiovascular pathology.

## 6 Conclusion

This review has highlighted the significant impact of cardiovascular comorbidities on the progression and outcomes of MS. The presence of CVDs is associated with increased disability, cognitive decline, and adverse radiological findings such as higher lesion burden and brain atrophy. Given the detrimental effects of CVDs on MS, proactive management of these comorbidities is crucial for improving neurological outcomes and overall quality of life. This paper has emphasized the importance of a neurocardiological approach to MS care, advocating for a multidisciplinary strategy incorporating cardiovascular risk assessment, preventative measures, and targeted interventions. Early detection and treatment of CVDs in MS patients, especially considering the relatively young age of this population, can lead to a cost‐effective, long‐term improvement in patient care.

Further research is needed to promote the integration of neurocardiology into standard MS care. Several promising areas for future investigation include evaluating the cost‐effectiveness of comprehensive neurocardiological protocols and assessing the impact of treating cardiovascular comorbidities on MS outcomes.

## Author Contributions

**Nicoleta Gherghel‐Pavăl:** conceptualization, investigation, methodology, visualization, data curation, writing – review and editing, writing – original draft. **Denis Pavăl:** conceptualization, writing – review and editing, visualization, supervision. **Adina D. Stan:** conceptualization, writing – review and editing, validation, supervision. **Olga H. Orășan:** supervision, writing – review and editing. **Adela V. Sitar‐Tăut:** supervision, writing – review and editing. **Angela Cozma:** conceptualization, writing – review and editing, supervision, validation.

## Conflicts of Interest

The authors declare no conflicts of interest.

## Transparency Statement

The lead author Nicoleta Gherghel‐Pavăl affirms that this manuscript is an honest, accurate, and transparent account of the study being reported; that no important aspects of the study have been omitted; and that any discrepancies from the study as planned (and, if relevant, registered) have been explained.

## Data Availability

Data sharing is not applicable to this article as no datasets were generated or analyzed during the current study.
